# Mangiferin Has an Additive Effect on the Apoptotic Properties of Hesperidin in *Cyclopia sp.* Tea Extracts

**DOI:** 10.1371/journal.pone.0092128

**Published:** 2014-03-14

**Authors:** Rafal Bartoszewski, Anna Hering, Marcin Marszałł, Justyna Stefanowicz Hajduk, Sylwia Bartoszewska, Niren Kapoor, Kinga Kochan, Renata Ochocka

**Affiliations:** 1 Department of Biology and Pharmaceutical Botany, Medical University of Gdansk, Gdansk, Poland; 2 Department of Inorganic Chemistry, Medical University of Gdansk, Gdansk, Poland; 3 Department of Neurology, University of Alabama at Birmingham, Birmingham, Alabama, United States of America; 4 Department of Toxicology, Medical University of Gdansk, Gdansk, Poland; School of Medicine, University of Belgrade, Serbia

## Abstract

A variety of biological pro-health activities have been reported for mangiferin and hesperidin, two major phenolic compounds of Honeybush (*Cyclopia sp.)* tea extracts. Given their increasing popularity, there is a need for understanding the mechanisms underlying the biological effects of these compounds. In this study, we used real-time cytotoxicity cellular analysis of the *Cyclopia sp*. extracts on HeLa cells and found that the higher hesperidin content in non-fermented "green" extracts correlated with their higher cytotoxicity compared to the fermented extracts. We also found that mangiferin had a modulatory effect on the apoptotic effects of hesperidin. Quantitative PCR analysis of hesperidin-induced changes in apoptotic gene expression profile indicated that two death receptor pathway members, TRADD and TRAMP, were up regulated. The results of this study suggest that hesperidin mediates apoptosis in HeLa cells through extrinsic pathway for programmed cell death.

## Introduction

The genus *Cyclopia* (Fabaceae family) is endemic to South Africa and consists of approximately 24 species. Leaves of several *Cyclopia* species are used to brew a traditional herbal tea that is known as honeybush tea [1], [2]. Honeybush tea, besides being a popular beverage in South Africa, is growing in popularity worldwide. Therefore, it is important to evaluate the honeybush effects on the human body. The major phenolic compounds present in *Cyclopia sp*. plant material are xanthones and their C-glucosides that include mangiferin and isomangiferin, and the flavanone glycoside hesperidin (hesperetin 7- O-rutinoside) [3]. Mangiferin and hesperidin have been reported to have anti-oxidative, anti-carcinogenic (cytotoxic), and anti-inflammatory properties. Therefore, the therapeutic properties of these natural phenolic compounds, particularly with regard to the anti-carcinogenic properties, have been of great research interest.

Hesperidin, originally found in citrus fruits [4], induces cell growth arrest and apoptosis in many cancer cell lines, including colon, breast and pancreatic cancer cells [5]–[8]. However, the molecular mechanisms underlying these cytostatic and cytotoxic effects of hesperidin remain to be fully understood. In human colon cancer cells, hesperidin has been shown to promote capsase-3 activation [6]. Recent studies indicate that hesperidin recruits a member of the ligand-dependent nuclear receptor superfamily: peroxisome proliferator-activated receptor-gamma (PPAR-γ) to induce it’s biological effects [8]. PPAR-γ was shown to be responsible for regulating cellular proliferation and differentiation by inducing apoptosis in a wide spectrum of human tumor cell lines [9], [10]. Furthermore, hesperidin-promoted protein 53 (p53) accumulation and down-regulated constitutively-active NF-kB that led to apoptosis in NALM-6 cells [8].

The other main phenolic compound of *Cyclopia sp.,* mangiferin, exhibits a wide range of biological activities in addition to its anti-tumor effects. Mangiferin has been shown to exhibit anti-diabetic [11], antiviral [12], [13], immunomodulatory [14], radioprotective [15], hepatoprotective [16], anti-inflammatory [17], vasodilatory effects [18], and antiplatelet [19] activities under different experimental conditions. Mangiferin also has effects on metalloproteinase-7 (MMP-7) and -9, the epithelial–mesenchymal transition (EMT), the β-catenin pathway, and inhibits proliferation and suppresses the migration and invasion of breast cancer cells [20]. Recent studies in the human acute myeloid leukemia cell line HL-60 showed that mangiferin induces apoptosis by suppressing entry of nuclear NF-kB [21]. Despite these numerous reports, the molecular mechanism of these compounds, especially regarding their combined effects, requires further clarification.

In this study, we performed real-time cellular analysis of cytotoxity of honeybush tea (*Cyclopia sp.*) extracts on the human cervix adenocarcinoma (HeLa) cell line. We found that the non-fermented "green" extracts exhibited higher cytotoxicity, which may correlate with its higher hesperidin content. We also found that the presence of mangiferin enhances the apoptotic effect of hesperidin. Furthermore, hesperidin treatment induces expression of the TNFR1 associated death domain protein (TRADD) and TNF receptor superfamily 25 (TNFRSF25 also known as TRAMP), suggesting that hesperidin mediates apoptosis in HeLa cells through activation of the extrinsic pathway of programmed cell death.

## Materials and Methods

### Materials

Mangiferin (MW 422.34 g/mol), hesperidin (MW 610.560 g/mol), tetrazolium bromide (MTT), Dulbecco’s modified Eagle’s medium (DMEM), PBS, and DMSO were purchased from Sigma Chemical Co. (St. Louis, MO, USA). For cell culture experiments, stock solutions of mangiferin (0.5 mg/ml) and hesperidin (0.12 mg/ml) in 98% ethanol were used.

### Plant material and extract preparation

The dried, fermented *Cyclopia genistoides* material was a gift from KAWON - HURT Co. (Gostyn, Poland). The non-fermented (“green”) *Cyclopia intermedia* material were purchased from Ukajali Marcin Majka Co. (Krakow, Poland). The resulting extracts and solvents used are listed in Table 1. Representative samples of each plant material were deposited at Department of Biology and Pharmaceutical Botany, Medical University of Gdansk, Poland. Plant material consisted of both stems and leaves. Briefly, following mechanical homogenization, a 15 g portion of dry, plant material (particle size < 2.0mm) was exhaustively extracted for 30 minutes in 90°C with 100 ml of water/ethanol (as specified in Table 1), using an ultrasonic water bath (50 Hz (3x for 30 min.)). The obtained extracts were subsequently passed through Whatman filter paper (390 μm pore size) and evaporated under vacuum at 60°C. The resulting dry residue was redissolved at 80°C in 25 ml of water or 50% (v/v) EtOH and stored at 4 °C. The extract's mangiferin and hesperidin contents were analyzed using HPLC with coulometric electrochemical and diode array spectrophotometric detections.

**Table 1:** Summary of used *Cyclopia sp extracts* and solvents.

Cyclopia sp. extract	Type of plant material	Solvent
**C**	non-fermented (“green”)	H_2_O
**D**	Fermented	H_2_O
**E**	non-fermented (“green”)	50% (v/v) EtOH
**F**	Fermented	50% (v/v) EtOH

### Chromatography

The HPLC system consisted of spectrophotometric diode array 340S detector pump P 580, an automated sample injector ASI-100, and column thermostate STH 585, all from Dionex Corporation (Sunnyvale, CA, USA), and a Coulochem II electrochemical detector – equipped with a 5020 model guard and a 5010 model analytical cell (ESA, Chelmsford, MA, USA) operated by the Chromeleon chromatography-management system (version 6.8 (Dionex)). Compounds were separated on a Hypersil Gold C_18_ column (150 mm×4.6 mm I.D., 5 μm particle) with Hypersil Gold guard column (10 mm×4.6 mm I.D., 5 μm particle), both from Thermo Electron Corporation (Dreieich, Germany). The isocratic mobile phase was 15 mM sodium phosphate, pH 4.0 with 85% orthophosphoric acid and acetonitrile (65/35 v/v). The flow rate was 1.0 ml min^−1^. The mobile phase was filtered through a 0.22-μm membrane filter and vacuum degassed before use. The injection volume was 20 μl. The column and automated sample injector thermostats were set at 20 and 8°C, respectively. The electrochemical behavior of mangiferin and hesperidin were studied by repeated injection of working standard solutions (100 μg/ml) and by detection at potentials from –0.5 to +1.2 V [22]. Hydrodynamic voltammograms of the analytes exhibited good responses in the ranges from +0.35 to +0.95 V. The potentials applied were: guard cell +1.1 V, first working electrode (E1) +0.35 V, and the second working electrode (E2) +0.95 V. Detection was confirmed by a photodiode array detector at 225, 254, 280 and 360 nm wavelengths, respectively [23]. The calibration curves for the determination of all analyses showed good linearity over the investigated ranges (10.0 – 100.0 ng, R2 > 0.9995). The limit of detection (LOD) and limit of quantification (LOQ) for mangiferin and hesperidin were: 0.35, 9.7 and 0.24, 8.6 ng/ml, respectively. The precision and reproducibility were evaluated by six replicated analyses, and the R.S.D. values were less than 0.9% and 3.7%. The recoveries were between 94.5 and 102.4%.

#### Cell lines and culture conditions

HeLa S3 and HaCaT (immortal human keratinocyte) cells were obtained from ATCC (www.atcc.org). HeLa and HaCaT cells were cultured in DMEM (Sigma) with 10% FBS at 37°C in a humidified incubator at 5% CO_2_. For HeLa cells medium was supplemented with 2 mM L-glutamine. HaCaT cells were maintained until passage 10. Cells were allowed to grow until 70–80% confluent prior to experiments.

### Cell viability assays

#### xCELLigence System

For real - time monitoring of cell viability, we used the Roche xCeligence system. The growth profiles of each cell type were first generated to obtain the optimal time for adding the extract/compound being tested (at 50% confluence). Briefly, HeLa and HaCAT cells (2000 cells per well) were seeded in the 16 wells PC plates, 24h prior to the treatment. Control cells were cultured in a presence of water or EtOH vehicle. Treated cells were incubated with extracts (from 0.01 to 50 μg/ml final concentration) for the next 24h, and every 15 minutes cell conductances were recorded. All experiments were performed in duplicate, in 3 independent repeats. Similar assays were performed for the compounds. For combined treatments, mangiferin was used at constant concentration of 5 μg/ml, while hesperidin was added at different concentrations. Roche RTCA software v. 1.2.1 was used to calculate the half maximal inhibitory concentration (IC50) values. As a reference control, the IC50 value of vinblastine sulfate was assayed in HeLa cells.

#### MTT Assays

of cell viability were performed in 96 well plates. One day after plating 500 cells per well, different concentrations of hesperidin and mangiferin were applied. For the combined treatments, mangiferin was used at constant concentration 5 μg/ml, while hesperidin was added at different concentrations. After a 24 hour incubation with the specified compounds, medium containing 1 mg/ml MTT was added to cells for a final concentration of 0.5 mg/ml and incubated at 37°C for 4 h. The medium was aspirated, and the formazan product was solubilized with DMSO. The absorbance at 630 nm (background absorbance) was subtracted from absorbance at 570 nm for each well. There were 6 replicates for each tested concentration. All experiments were repeated at least twice. The resulting IC50 values were calculated with GraFit 7 software [24].

#### Trypan blue exclusion assays

Following 48 after siRNA transfection, cells were treated as specified and subsequently collected (including floating cells), stained with 0.2% trypan blue (Sigma-Aldrich) and counted using a Countess Automated Cell Counter. The results were expressed as percentage of live control cells, which is represented as 100%. All the experiments were performed in triplicate at least two times.

### Hoechst 33342 staining

Apoptotic and necrotic cell death were characterized by the use of Hoechst 33342 staining (Molecular Probes). Cells were stained with 0.5 μg/ml Hoechst 33342 for 30 min at 37°C. After washing the cells twice with phosphate buffered saline, the cells were examined under UV light with a digital camera attached to a fluorescence microscope. Apoptotic cells were characterized by fragmented and/or condensed nuclei and necrotic cells by diffuse and irregular nuclei.

### DNA isolation and agarose gel electrophoresis

Treated cells were lysed in 50 mM Tris-HCl, pH 7.5, containing 20 mM EDTA and 1% NP40. After lysis, the cell debris was removed by centrifugation at 5000 g for 10 min. DNAse-free RNAse (Sigma-Aldrich) was added to the lysates at a final concentration of 5 μg/ml and SDS was added at final concentration of 1%, and the lysates were then incubated for 2 h at 56°C. Next, proteinase K (Sigma-Aldrich) was added to the RNAse-treated lysates to a final concentration of 2.5 μg/ml. The lysates were further incubated for 2 h at 37°C. DNA was precipitated with 3M ammonium acetate (final) and ethanol overnight at −80°C. After centrifugation and drying, the DNA was dissolved in TE-buffer (10 mM Tris, pH 8.0, containing 1 mM EDTA). Agarose gel electrophoresis of DNA was performed in 2.0% agarose gels containing 0.5 μg/ml ethidium bromide. To visualize apoptotic alterations to DNA integrity, the DNA bands were observed on a UV transilluminator. The experiments were performed in triplicate twice.

### Isolation of cellular RNA

Total cellular RNA was isolated using mRNeasy (QIAGEN). The RNA concentration was calculated based on the absorbance at 260 nm. RNA samples were stored at –70°C.

### Real time PCR and gene expression analysis

cDNA was obtained with Maxima First Strand cDNA Synthesis Kit for RT-qPCR (Thermo Scientific).

#### Real time PCR gene expression arrays

cDNA obtained from cells treated with hesperidin alone and in combination with mangiferin, as well as from cells treated with control vehicle, were applied on The Applied Biosystems TaqMan Array Human Apoptosis 96-well Plates (INVITROGEN 4414072). Each plate contains 88 assays for apoptosis associated genes and 4 assays for candidate endogenous control genes. The PCR reactions were set according to manufacturer's instructions and performed on ABI7500 real time system. The resulting data were analyzed with ABI 2.05 software based on the comparative dCT method [25]. To validate arrays results, we independently analyzed (with RT-PCR) all significantly changed transcripts (RQ above 2 or below 0.5) as well as 30 genes with unaffected levels.

#### Measurement of expression using quantitative Real Time PCR (qRT-PCR)

We used SybyrGreen RT-PCR Master MixReagents (Applied Biosystems) and followed the manufacturer’s protocol (Relative Quantification; Applied Biosystem 7300/7500 Real Time PCR System; 2004). The SybyrGreen PCR primers sequences are combined in the Table S1. For all SybyrGreen primers, melting curve analyses were performed. The resulting data were analyzed with ABI 2.05 software based on comparative dCT method. To further confirm expression of specified genes, we used specific TaqMan probes (BIRC7 assay id Hs01086675_m1; BAX assay id Hs00180269_m1; BCL2 assay id Hs00608023_m1; GAPDH assay id Hs02758991_g1; 18S rRNA assay id Hs99999901_s1) and TaqMan One-Step RT-PCR Master MixReagents (Applied Biosystems) as described previously [26]. The resulting data were analyzed with ABI 2.05 software based on the comparative relative standard curve method [27].

### TRADD siRNA transfection

For TRADD silencing, we used Ambion Silencer Pre-designed siRNA (assay id: s16607, the siRNA sense sequence was 5'-GCGCAUACCUGUUUGUGGAtt-3' and antisense sequence was 5'-UCCACAAACAGGUAUGCGCtg-3') and corresponding Ambion siRNA Negative Control 1 (Invitrogen cat. no. 4390843, the siRNA sequence is Ambion proprietary information). The HeLa cells were transfected using the liposome Lipofectamine RNAiMax (Invitrogen) according to manufacturer's protocol. The transfected cells were cultured for 2 days prior to further analysis. The degree of TRADD knockdown was identified by quantitative RT-PCR.

### Western Blots

Cells were lysed in RIPA buffer (150 mM NaCl, 1% NP-40, 0.5% sodium deoxycholate, 0.1% SDS, 50 mM Tris- HCl, pH 8.0) supplemented with protease Inhibitor Complete Mini (Roche) on ice for 15 min. The cell lysates were rotated at 4°C for 30 min and the insoluble material was removed by centrifugation at 14,000 rpm for 15 min. Protein concentrations were determined by BioRad Protein Assay (BioRad) using bovine serum albumin (BSA) as a standard. Following the normalization of protein concentrations, lysates were mixed with an equal volume of 2X Laemmli sample buffer and incubated for 5 min at 95°C prior to separation by SDS PAGE on stain free TGX gradient gels (BioRad). Following SDS PAGE, the proteins were transferred to polyvinylidene fluoride membranes (300 mA for 90 min at 4°C). The membranes were then blocked with BCA (SIGMA) proteins dissolved in PBS/Tween-20 (3% BCA, 0.5% Tween-20 for 1–2 h), followed by immunoblotting with the primary antibody specified for each experiment (Bax abcam ab32503; BCL2 abcam ab59348, beta actin abcam ab1801, and caspase 8 santa cruz sc-81661). After the washing steps, the membranes were incubated with goat anti-rabbit IgG (H+L) or with goat anti-mouse IgG (H+L) HRP-conjugated secondary antibodies (BioRad) and detected using ECL (Pierce). Densitometry was performed using Image Lab software v. 4.1 (BioRad).

### Statistical analysis

Results were expressed as means ± standard deviations (SD). Statistical significance among means was determined using the Student’s t-test (two samples, paired and unpaired).

## Results

### Real-time cellular analysis of Cyclopia sp. extracts cytotoxicity

**Figure 1:**
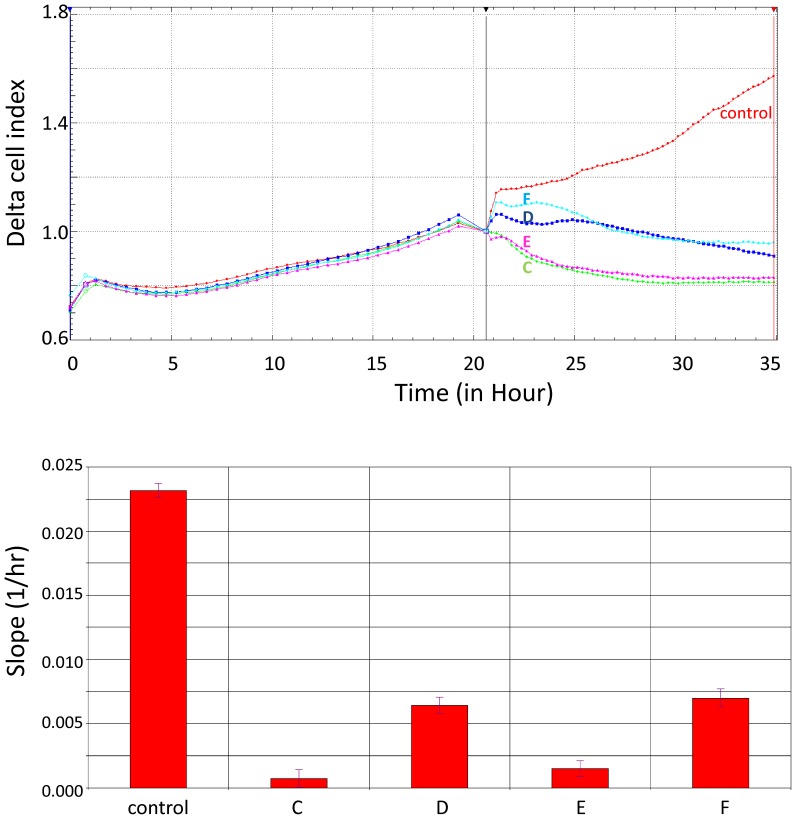
Real-time cellular analysis of Cyclopia sp. extracts on cell growth curves. Impact of extract treatment (5 μg/ml final concentration for each extracts) on HeLa cells growth curves is presented in the top panel. Each curve represents an average of 3 replicates from 2 independent experiments. The bottom panel represents changes in cell proliferation during 12h treatment expressed as the growth curve slope. The error bars represent the standard derivation values.

The *xCELLigence* RTCA technology is recognized as an accurate technique for non-invasive detection of cell viability and motility [28]–[33]. Although this technical approach strongly correlates with conventional methods, RTCA technology shows increased sensitivity. Thus, to determine impact of studied extracts on cervical cancer cells (HeLa) proliferation, as well as to validate if "green" and fermented material have different cytotoxic properties, we utilized this method. Furthermore, we compared solvent effects on cell proliferation (H_2_O or EtOH 50% (v/v)), for both fermented and non-fermented extracts. All extracts exhibited their cytotoxic effects up to 12h after addition. The most pronounced inhibition of HeLa cells proliferation was observed after applying "green" origin extracts (**C** and **E** Table 1) regardless of solvent (*Figure 1*). Next, we accessed IC50 values after 12h of treatment for each studied extract as shown in Table 2. For "green" **C** and **E** extracts, the recorded IC50 values were 1.458 μg/ml and 1.667 μg/ml, respectively. For fermented **D** and **F** (Table 1) extracts, the recorded IC50 values were 13.30 μg/ml and 10.60 μg/ml, respectively. In order to evaluate potential anti-cancer potential of these extracts, we performed similar analyses of their effects on control cells: immortalized human keratinocytes.

**Table 2:** IC50 values of studied Cyclopia sp. extracts, calculated with RTCA.

Cyclopia sp. extract	HeLa IC50 and R^2^	HaCaT IC50 and R^2^
**C**	1.458 μg/ml; R^2^ = 0.946	6.76 μg/ml; R^2^ = 0.921
**D**	13.30 μg/ml; R^2^ = 0.978	18.45 μg/ml; R^2^ = 0.937
**E**	1.667 μg/m; R^2^ = 0.981	5.26 μg/m; R^2^ = 0.9876
**F**	10.60 μg/ml; R^2^ = 0.993	17.38 μg/ml; R^2^ = 0.945

IC50 values were calculated in RTCA software, with use of non-linear regression (sigmoidal dose response model). *Each extract concentration was tested in triplicate and experiments were repeated twice.*

**Figure 2:**
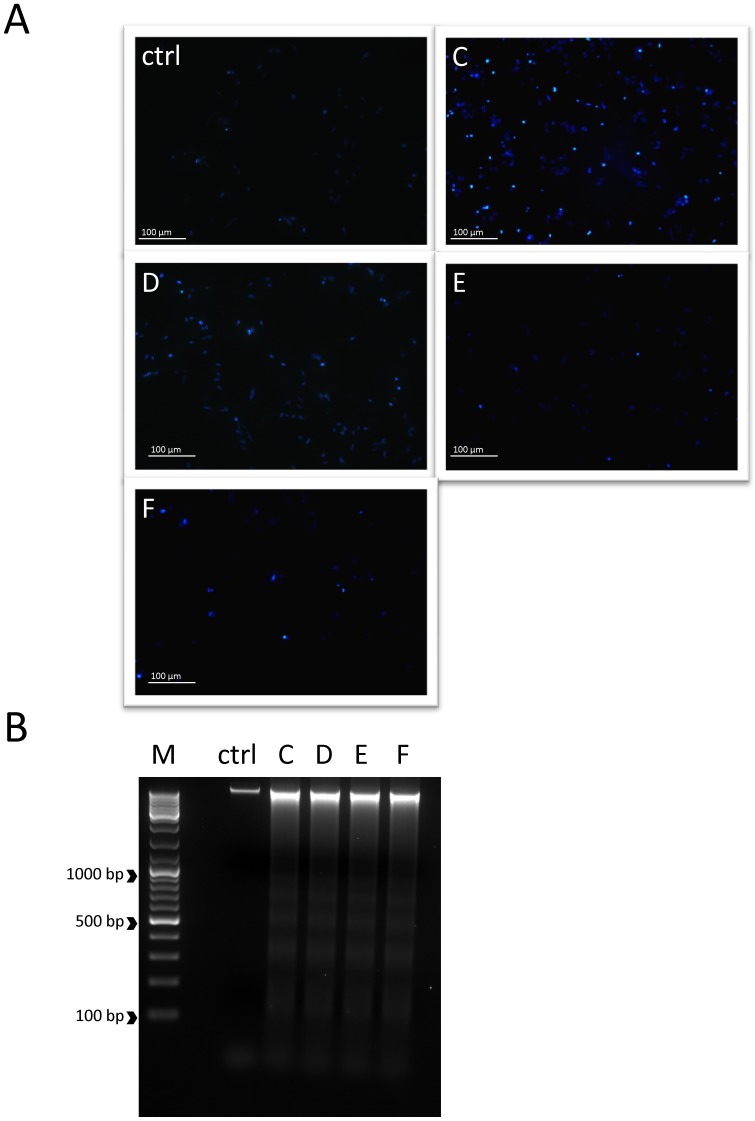
Cyclopia sp. extracts induce apoptotic changes in HeLa cells. **A**) Hoechst 33342 staining was used to detect the formation of condensed pycnotic nucleus. The cells were incubated in a presence of extracts (at concentrations equal to IC50 values) for 12h; **B**) Characterization of DNA fragmentation induced by extracts (at concentrations equal to IC50 values) was examined by agarose gel electrophoresis. In untreated cells (ctrl), there was no DNA fragmentation. Under treatment with the different extracts, DNA fragmentation occurs (M - DNA standard, Fermentas SM0333).

As presented in Table 2, all extracts impaired HeLa cell proliferation more significantly than HaCaT. IC50 values recorded for the HaCaT cells treated with the extracts were significantly higher than those recorded for HeLa. Similarly, the "green" extracts exhibited higher cytotoxity in the HaCaT cells.As shown in *Figure 2* all extracts induced apoptosis-related DNA fragmentation and the formation of condensed pycnotic nuclei in HeLa cells.

### Mangiferin and hesperidin content in the extracts

**Figure 3:**
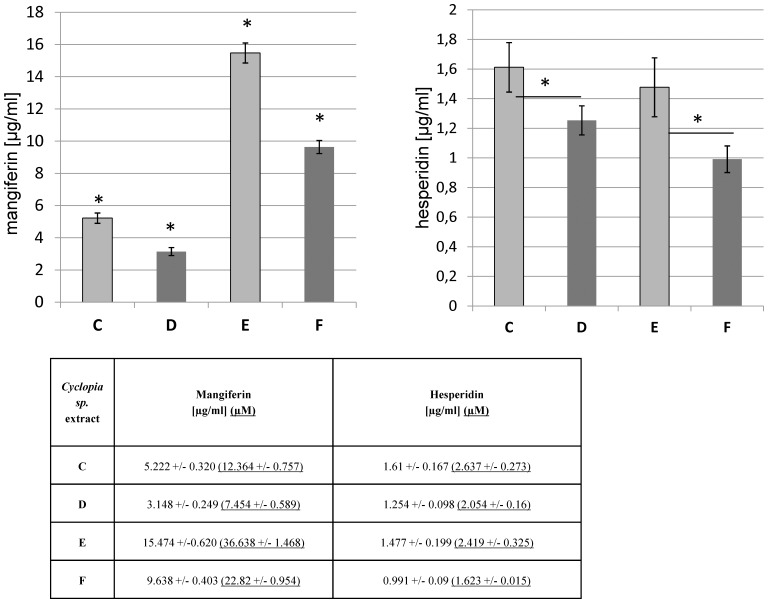
Mangiferin (left panel) and hesperidin (right panel) content in Cyclopia sp. extracts. The "green" extracts (C and E) are marked in grey while the fermented extracts are marked in black. Error bars represent standard derivations. Significant differences (p value < 0.05) in compound content are marked with an *.

Since hesperidin and mangiferin are main phenolic compounds in *Cyclopia sp*. extracts, we analyzed their amounts in studied extracts with HPLC with Photodiode Array Coulometric and Electrochemical Array Detection (*Figure 3*). Despite individual differences, mangiferin content was significantly higher (3 to 5 fold) in both, non- and fermented ethanolic extracts (**E** and **F**) than in water based one (**C** and **D**). The hesperidin was present in significantly or slightly higher amounts in "green" extracts (**C** and **E,** respectively). Those data suggest that the higher hesperidin content could be responsible for lower IC50 values recorded for "green" extracts.

### Real-time cellular analysis of mangiferin and hesperidin cytotoxicity

**Figure 4:**
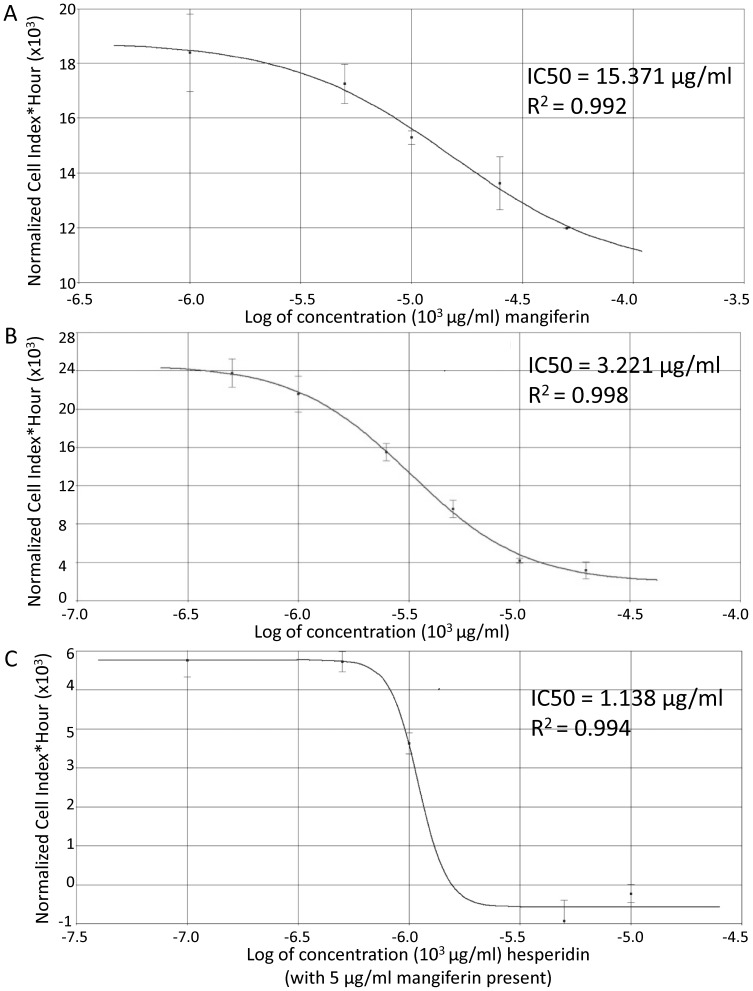
RTCA measurement of IC50 value of A) mangiferin, B) hesperidin and C) hesperidin in the presence of 5 μg/ml mangiferin in HeLa cells, after 12h of treatment. Error bars represent the standard derivations. IC50 values were calculated in RTCA software with use of non-linear regression (sigmoidal dose response model). Each extract concentration was tested in triplicate and experiments were repeated twice. Obtained IC50 values were validated with the MTT method in independent experiments (HeLa, 12h exposure). MTT recorded IC50 values were 4.210 +/–0.320 μg/ml for hesperidin, 20.087 +/–0.320 μg/ml for mangiferin, and 1.623 +/– 0.323 μg/ml for hesperidin in presence of mangiferin (5 μg/ml).

Although the type of material tested (green or fermented), the solvent, and the mangiferin content did not have a significant impact on cell proliferation, we analyzed cytotoxic properties of both mangiferin and hesperidin. As shown on *Figure 4*, the IC50 value of hesperidin was 3.22 μg/ml (5.275 μM) recorded after 12 h in HeLa cells. Analogically, the registered IC50 value for mangiferin is 15.37 μg/ml (36.39 μM).

Since the recorded IC50 values for the extracts were mostly lower than those of hesperidin and mangiferin alone, we decided to test combined effects of those substances on cells proliferation. Hesperidin content was higher in green extracts, and this substance itself displayed a lower IC50 value. Thus, we decided to test effects of hesperidin on cell proliferation in presence of relatively low constant concentration of mangiferin 5 μg/ml (11.84 μM ), which did not have a significant impact on cell survival.

As shown in *Figure 4*, in a presence of mangiferin the cytotoxic effects of hesperidin are much more pronounced and IC50 value drops to 1.138 μg/ml (1.86 μM) after 12h in HeLa cells. Thus, cytotoxic effects of hesperidin in a presence of mangiferin were similar to those observed for "green" extracts.

### Hesperidin increases TRADD mRNA levels in HeLa cells leading to the activation of the extrinsic apoptosis pathway

**Figure 5:**
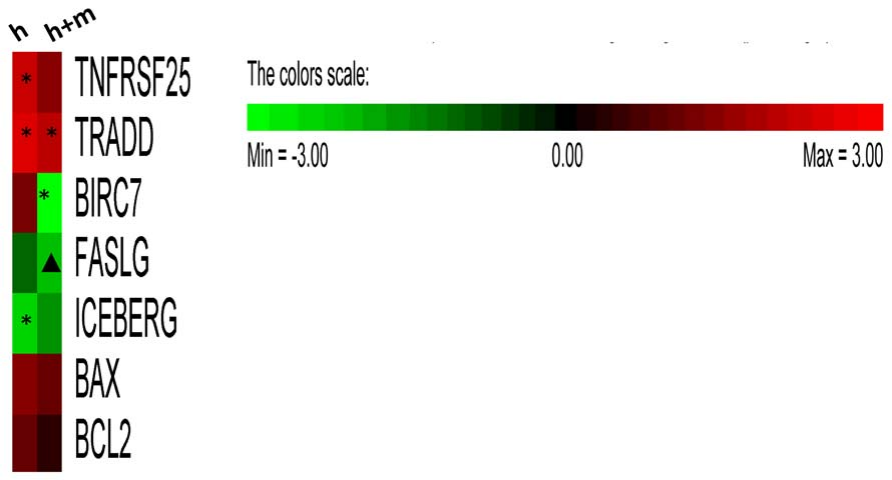
Representation of validated PCR apoptotic gene expression array results. The heat map left column marked **h** and **h+m,** represents relative expression fold changes (relative to 18S, GAPDH, HRTP1 and GUSB) versus EtOH control) in HeLa treated with hesperidin **(h)** (2.5 μg/ml, 4.01 μM) and hesperidin (2.5 μg/ml, 4.01 μM) in a presence of mangiferin (5 μg/ml, 11.84 μM) **(h+m)**, respectively. HeLa cells were incubated for 12h in presence of EtOH (EtOH concentration was adjusted to combined treatment conditions) or specified compounds. "*" denotes relative fold change ≤ –2 and ≥2;"▴" denotes relative fold change ≤ –1.5 and ≥1.5. For heat map generation RQ values were converted to fold change and PermutMatrix software was used [49].

In order to examine changes in apoptotic gene expression induced by hesperidin alone and in a presence of mangiferin, we performed PCR array-based analysis in HeLa cells. Among the examined 92 genes, hesperidin alone significantly influenced the expression of 3 genes (2 up regulated and 1 down regulated) (*Figure 5*). The presence of mangiferin resulted in down regulation of one gene and up regulation of another (*Figure 5*).

**Figure 6:**
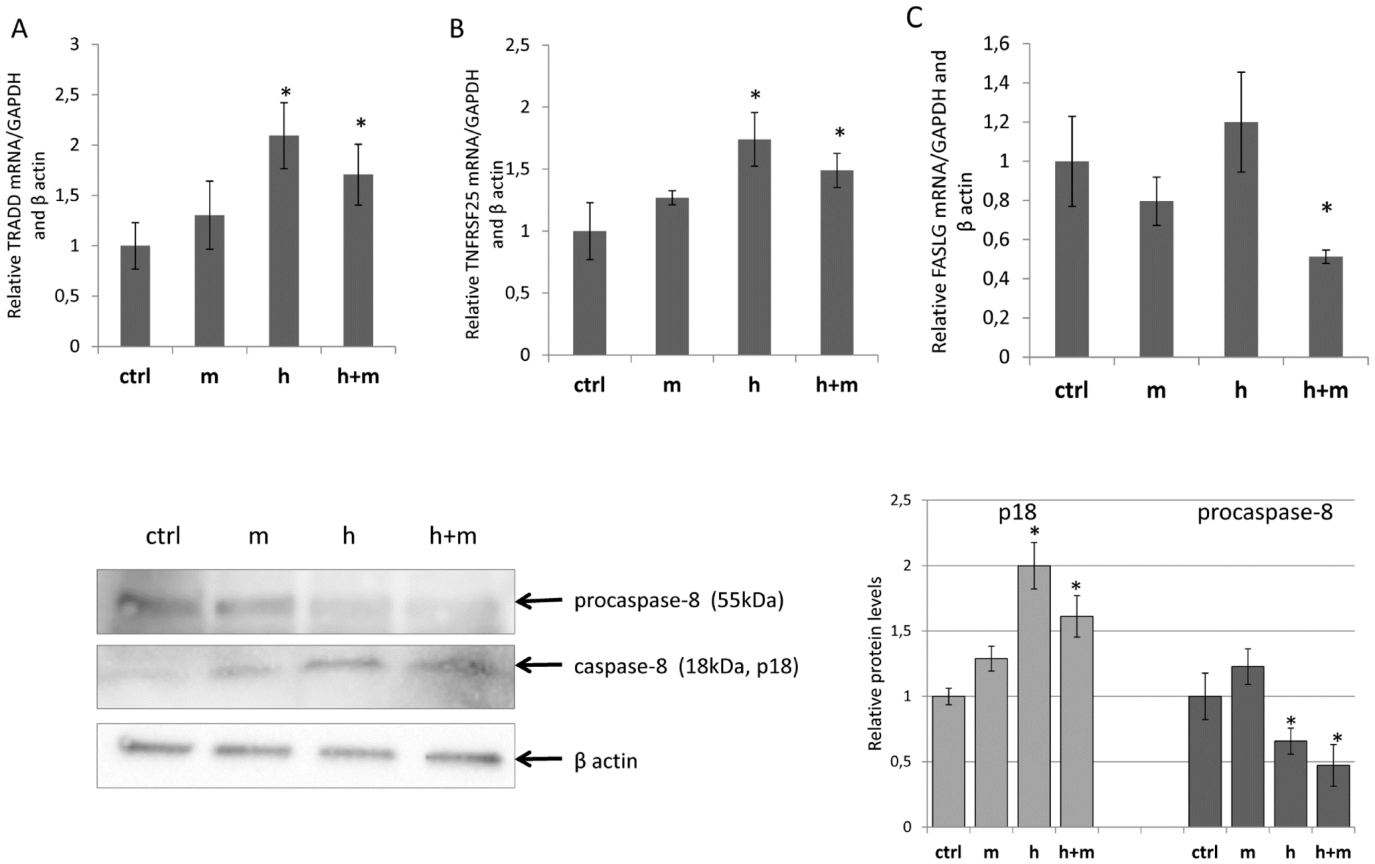
Hesperidin treatment induced changes in mRNA levels of death receptors apoptosis pathway members A) TRADD, B) TNFRSF25, and C) FASLG. Following 12h treatment of HeLa cells with hesperidin (h) (2.5 μg/ml, 4.01 μM), mangiferin (5 μg/ml, 11.84 μM) (m), and hesperidin (2.5 μg/ml, 4.01 μM) in a presence of mangiferin (5 μg/ml, 11.84 μM) (h+m), the mRNA levels were monitored in real - time PCR experiments. The results from 2 independent experiments (n = 8) are plotted relative to glyceraldehyde-3-phosphate dehydrogenase (GAPDH) and beta actin mRNA levels and expressed as a fold-change over the EtOH control. SybyrGreen primer sequences are listed in Table S1. Error bars represent standard derivations. Significant (p<0.05) changes are marked with an "*". **Bottom panel**: following 12h of treatment of HeLa cells with hesperidin (h) (2.5 μg/ml, 4.01 μM), mangiferin (5 μg/ml, 11.84 μM) (m), and hesperidin (2.5 μg/ml, 4.01 μM) in a presence of mangiferin (5 μg/ml, 11.84 μM) (h+m), the protein levels of procaspase-8 (55 kDa) and active caspase-8 p18 subunit (p18) were detected by Western Blot. Two individual samples (7 μg of total protein per lane) were tested for each treatment and the experiments were repeated three times. Protein levels were related to the control sample. Error bars represent standard derivations. Significant (p<0.05) changes are marked with an "*".

**Figure 7:**
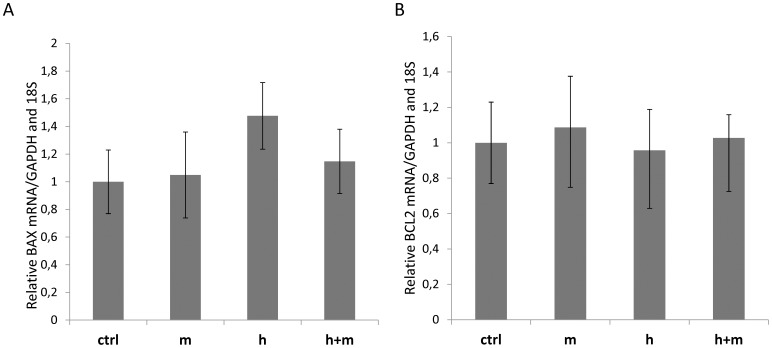
Hesperidin and mangiferin treatment induced changes in mRNA levels of the anti-apoptotic genes A) ICEBERG and B) BIRC7. Following 12h treatment of HeLa cells with hesperidin (h) (2.5 μg/ml, 4.01 μM), mangiferin (5 μg/ml, 11,84 μM) (m), and hesperidin (2.5 μg/ml, 4,.01 μM) in the presence of mangiferin (5 μg/ml, 11.84 μM) (h+m), the mRNA levels were monitored in real - time PCR experiments. For ICEBERG mRNA levels, the results from 2 independent experiments (n = 8) are plotted relative to glyceraldehyde-3-phosphate dehydrogenase (GAPDH) and beta actin mRNA levels and expressed as a fold-change over the EtOH control. SybyrGreen primer sequences are listed in Table S1. For BIRC7 mRNA levels, the results from 2 independent experiments (n = 8) are plotted relative to glyceraldehyde-3-phosphate dehydrogenase (GAPDH) and 18S rRNA levels and expressed as a fold-change over the EtOH control. Error bars represent standard derivations. Significant (p<0.05) changes are marked with an "*".

We validated our array results with real-time PCR. The main observed transcriptional effect of 4 μM hesperidin treatment in HeLa cells was an increase of mRNA levels of members of death receptors apoptotic pathway: tumor necrosis factor receptor type 1-associated death domain protein (TRADD) and tumor necrosis factor receptor superfamily member 25 (TNFRSF25*)* also known as TRAMP *(* *Figure 6A and B* *)*. Furthermore, we observed a significant decrease in mRNA levels of the anti-apoptotic caspase-1 inhibitor iceberg (ICEBERG) (*Figure 7A*). In a presence of 12 μM mangiferin, TRADD mRNA levels were still significantly induced. However, induction of TNFRSF25 mRNA levels as well as reduction of ICEBERG mRNA levels were not as pronounced. With the addition of mangiferin, we observed down regulation of anti apoptotic bacculoviral IAP repeat-containing 7 (BIRC7) mRNA levels (*Figure 7*). Interestingly, the addition of mangiferin resulted in the down regulation of the mRNA levels of another member of death receptors apoptotic pathway, fas ligand (FASLG) (*Figure 6*). Although, we observed some elevation of BCL2-associated X protein (BAX) mRNA, this change was not significant during our 12h treatment We also examined effects of 12 μM mangiferin treatment in HeLa on mRNA the levels of TRADD, TNFRSF25, ICEBERG and FAS ligand (FASLG). As shown on *Figure 6* *and* *Figure 6A*, none of the mRNA levels were significantly affected by mangiferin alone. However, BIRC7 mRNA was significantly down-regulated by mangiferin treatment. However this mangiferin effect on BRIC7 mRNA was less pronounced than in the presence hesperidin (*Figure 7B*).

In order to examine if the hesperidin-related increase in expression of death receptor apoptotic pathway members leads to activation of this pathway, we followed caspase 8 activation. Hesperidin treatment alone and in presence of mangiferin resulted in activation of caspase 8 (both showing a reduction of procaspase and accumulation of active caspase), while mangiferin treatment had no effect on the activation of this caspase (*Figure 6*). Furthermore, neither PCR expression arrays nor real-time PCR analyses indicated changes in caspase 8 mRNA levels during the treatments.

**Figure 8:**
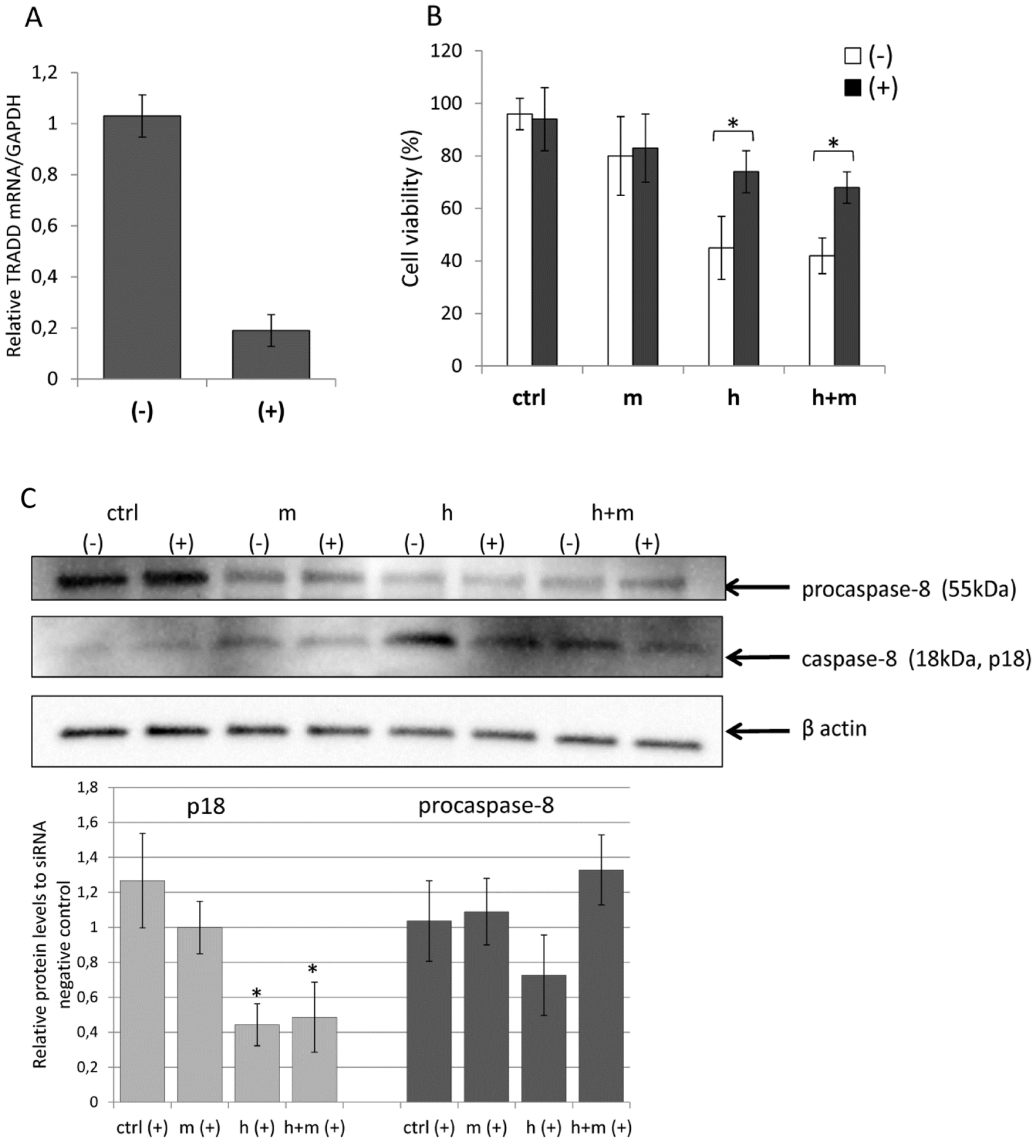
Silencing TRADD reduces active caspase-8 (p18) levels and increases HeLa cell survival. **Top panel: A**) The degree of TRADD knockdown was determined 48h after transfection with TRADD siRNA **(+)** or siRNA Negative Control **(**–**)** by quantitative RT-PCR. The TRADD mRNA levels results from 2 independent experiments (n = 8) are plotted relative to glyceraldehyde-3-phosphate dehydrogenase (GAPDH) and expressed as a fold change over the siRNA Negative Control. Error bars represent standard derivations. **B**) Following 48h after transfection with TRADD siRNA **(+)** or siRNA Negative Control **(-)** and 12h of treatment of HeLa cells with hesperidin (**h)** (2.5 μg/ml, 4.01 μM), mangiferin (5 μg/ml, 11.84 μM) (**m**), and hesperidin (2.5 μg/ml, 4.01 μM) in a presence of mangiferin (5 μg/ml, 11.84 μM) (**h+m**), the percentage of live cells were determined by trypan blue exclusion. The results were expressed as percentage of live control cells, which is represented as 100*%*. All the experiments were performed in triplicate at least two twice. Error bars represent standard derivations. Significant (p<0.05) changes are marked with an "*". **Bottom panel: C)** Following 48h after transfection with TRADD siRNA **(+)** or siRNA Negative Control **(**–**)** and 12h of treatment of HeLa cells with hesperidin (**h)** (2.5 μg/ml, 4.01 μM), mangiferin (5 μg/ml, 11.84 μM) (**m**), and hesperidin (2.5 μg/ml, 4.01 μM) in a presence of mangiferin (5 μg/ml, 11.84 μM) (**h+m**), the protein levels of procaspase-8 (55 kDa) and active caspase-8 p18 subunit (p18) were detected by Western Blot. Two individual samples (7 μg of total protein per lane) were tested for each treatment and the experiments were repeated three times. Protein levels were related to siRNA Negative Control Samples. Error bars represent standard derivations. Significant (p<0.05) changes are marked with an "*".

Since only TRADD expression was significantly induced by either hesperidin alone or hesperidin in the presence of mangiferin, we examined the effects of TRADD silencing on the activation of external apoptotic pathway. Although silencing TRADD did not totally eliminate caspase 8 activation and apoptosis during treatments, the silencing of TRADD resulted in lowering the active caspase 8 levels and in increasing cell survival (*Figure 8*).

**Figure 9:**
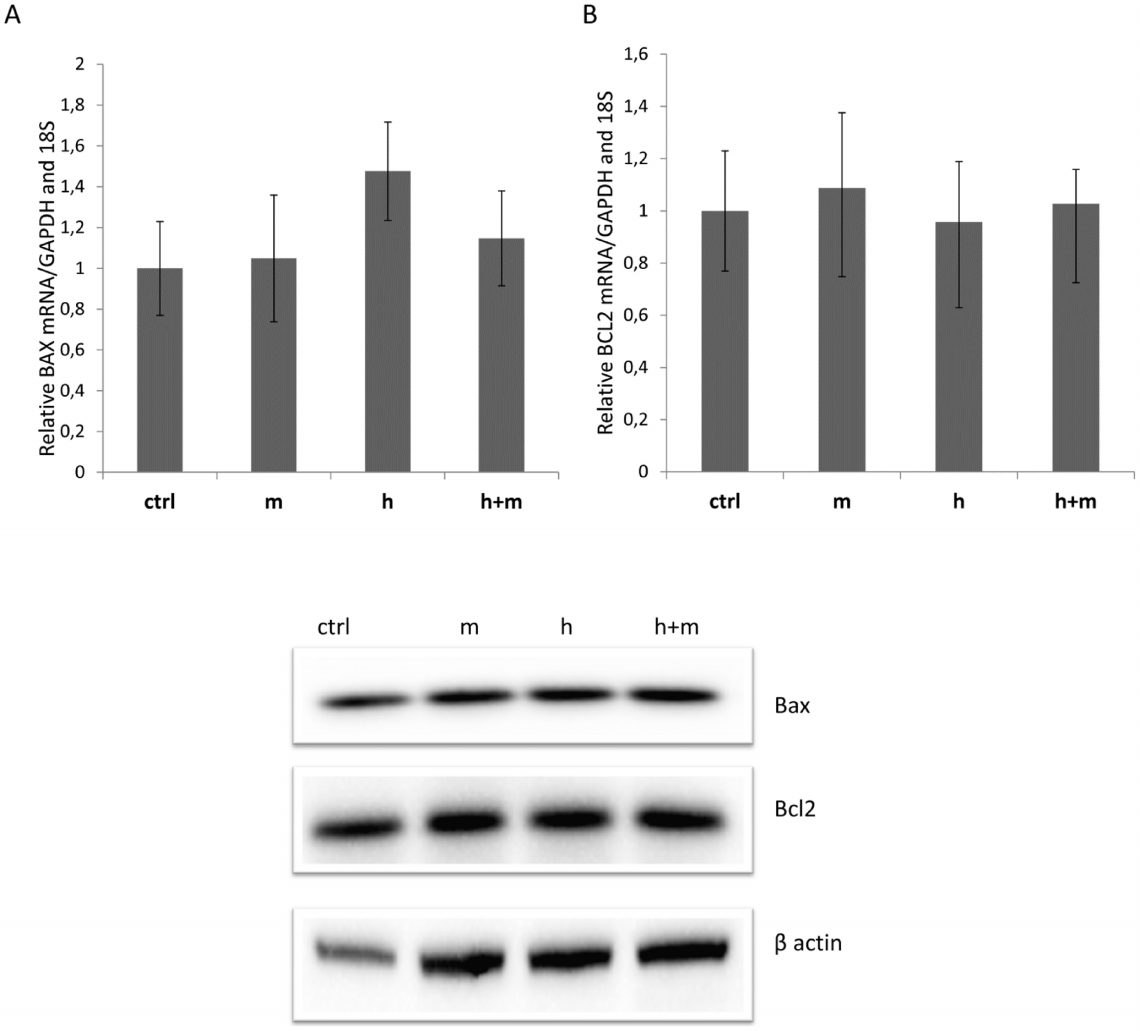
Hesperidin and mangiferin did not alter the expression of A) BAX and B) BCL2 during the 12h treatment. **Top panel:** Following 12h treatment of HeLa cells with hesperidin (**h)** (2.5 μg/ml, 4,01 μM), mangiferin (5 μg/ml, 11.84 μM) (**m**), and hesperidin (2.5 μg/ml, 4.01 μM) in a presence of mangiferin (5 μg/ml, 11.84 μM) (**h+m**), the mRNA levels were monitored in real - time PCR experiments. The BAX and BCL2 mRNA levels results from 2 independent experiments (n = 8) are plotted relative to glyceraldehyde-3-phosphate dehydrogenase (GAPDH) and 18S rRNA levels and expressed as a fold change over the EtOH control. Error bars represent standard derivations.**Bottom panel:** Following 12h of treatment of HeLa cells with hesperidin (**h)** (2.5 μg/ml, 4.01 μM), mangiferin (5 μg/ml, 11.84 μM) (**m**), and hesperidin (2.5 μg/ml, 4.01 μM) in a presence of mangiferin (5 μg/ml, 11.84 μM) (**h+m**), the protein levels of Bax and BCL2 were detected with SDS-PAGE and Western Blot and related to beta actin levels. 2 individual samples (3 μg of total protein per lane) were tested for each treatment and the experiments were repeated twice.

Since a BAX - related mechanism for hesperidin-induced apoptosis was proposed previously [5], we analyzed expression levels of this gene as well as of B-cell CLL/lymphoma 2 (BCL2) gene. BCL2 and BAX mRNA levels, as well as protein levels remain unaffected by all compounds (*Figure 9*) during 12h treatment in HeLa cells.

## Discussion

Honeybush (*Cyclopia sp*.) is well known for the production of teas and other food products. *Cyclopia* is also a good source of aqueous extracts and contains a variety of phenolic compounds including mangiferin and hesperidin. Since a variety of biological pro-health activities had been reported for mangiferin and hesperidin, it is important to understand the mechanisms underlying biological effects of these compounds. Furthermore, it is important that *Cyclopia* extract preparations are standardized according to their biological activity for both research and commercial applications.

In our studies, we tested the cytotoxic activities of different preparations of *Cyclopia* extracts in human cell lines, and the results indicate dramatic differences in the preparations. The green extracts inhibited cell proliferation the most and the solvent used did not appear to matter. Interestingly, the green non-fermented extracts had significantly higher hesperidin content than the fermented ones. Furthermore, mangiferin was detected at significantly higher levels in the fermented extracts.

In order to evaluate if hesperidin levels in *Cyclopia* extracts correlated with their pro-apoptotic activities, we analyzed impact of pure hesperidin and mangiferin on HeLa cell growth. Although the cytotoxic effects of hesperidin were more pronounced than those of mangiferin, the obtained IC50 values were significantly higher than recorded for *Cyclopia* "green" extracts. Thus, hesperidin nor mangiferin alone could not responsible for observed biological effects. Our data indicate that hesperidin in a presence of low mangiferin concentrations was significantly more cytotoxic than hesperidin alone. The IC50 values obtained for this combined treatment were close to the ones recorded for green extracts. These data suggest that mangiferin addition should be considered when testing other anticancer drugs. This idea is consistent with previous studies where mangiferin was used with oxaliplatin treatment and shown to improve the efficacy of this anti-cancer drug [34]. Although our studies indicated that the presence of mangiferin enhanced hesperidin cytotoxicity, we cannot exclude the possibility that other phenolic components of *Cyclopia* green extracts could account for some of these effects.

Interestingly, we did not observe an effect of hesperidin and mangiferin treatment on Bax or Bcl2 levels suggesting that the concentrations used were too low and/or of significant duration to induce Bax and Bcl2 mRNA and protein expression. We did, however, see significant changes in other genes. In our apoptotic gene expression analysis, we found that hesperidin increases the mRNA levels of members of extrinsic pathway for programmed cell death, (TRADD) and TNFRSF25 (TRAMP). TRADD is a signaling adaptor protein involved in extrinsic apoptosis that specifically interacts with an intracellular domain of death receptors. TRADD in turn triggers two opposite signaling pathways that lead to caspase activation for apoptosis induction and NF-kappaB activation for cell survival [35]–[37]. TRAMP (also known as TNFRSF25, WSL-1, LARD, TRAMP, Apo3) is member of the tumor necrosis factor (TNF) receptor family [38]. Overexpression of TRAMP leads to two major responses, NF-kB activation and apoptosis [39], [40]. TRAMP has been shown to interact directly with the adapter TRADD [38].

Among the tumor growth-inhibitory effects of mangiferin and hesperidin, the involvement of the NF-κB signaling pathway and suppression of NF-κB activation have been postulated [8], [41]–[46]. Thus, the observed increase of TRADD and TRAMP mRNA levels suggest that hesperidin affects the death receptor pathway, whereas NF-κB inactivation (driven by both mangiferin and hesperidin) triggers TRADD and TRAMP towards apoptosis. Furthermore, the hesperidin-related activation of extrinsic apoptosis pathway was confirmed by caspase-8 activation, while silencing TRADD expression resulted in higher survival of hesperidin treated cells and a corresponding decrease in active caspase-8 levels. Although we confirmed hesperidin induced an increase in mRNA levels of both TRAMP and TRADD, understanding the mechanism and consequences of this induction will require further studies. We also observed that mangiferin treatment caused reduction of BIRC7 that could sensitize the cells to death receptor apoptosis [47], [48]. Thus, the mangiferin-related reduction of BIRC7 could contribute to the enhanced hesperidin cytotoxicity. However, since mangiferin is also capable of influencing NF-kB activity, the role of BIRC7 down-regulation requires further study.

In summary, the major finding of this study is the integration of death receptors pathway in the apoptosis induced by the hesperidin in the HeLa cell line.

## Supporting Information

Table S1 **Primer pairs used in qPCR Sybyr Green experiments.**
(PDF)
