# Supplementary material for: Mangiferin Has an Additive Effect on the Apoptotic Properties of Hesperidin in Cyclopia sp. Tea Extracts
Source: PLoS One. 2014 Mar 14;9(3):e92128. doi: 10.1371/journal.pone.0092128 (PMC3954868; doi:10.1371/journal.pone.0092128)
Supplement: Table S1 — Primer pairs used in qPCR Sybyr Green experiments. (PDF) [file pone.0092128.s001.pdf]

Gene	Forward primer (5'-> 3')	Reverse primer (5'-> 3')
BAD	CCC AGA GTT TGA GCC GAG TG	CCC ATC CCT TCG TCG TCC T
BAX	TTC CGA GTG GCA GCT GAC AT	TTC CAG ATG GTG AGT GAG GC
BCL10	GTG AAG AAG GAC GCC TTA GAA A	TCA ACA AGG GTG TCC AGA CCT
BCL2	GGT GAA CTG GGG GAG GAT TGT	CTT CAG AGA CAG CCA GGA GAA
BCL2L2	GCG GAG TTC ACA GCT CTA TAC	AAA AGG CCC CTA CAG TTA CCA
beta actin	CAT GTA CGT TGC TAT CCA GGC	CTC CTT AAT GTC ACG CAC GAT
BID	ATG GAC CGT AGC ATC CCT CC	GTA GGT GCG TAG GTT CTG GT
BIK	GAC CTG GAC CCT ATG GAG GAC	CCT CAG TCT GGT CGT AGA TGA
BIP	GTT CTT GCC GTT CAA GGT GG	TGG TAC AGT AAC AAC TGC ATG
BIRC6	CAG CAG CTC TTA TCA GCA TGT	AAC TGT GGC CCA CTT AGC AAC
BIRC7	GCT CTG AGG AGT TGC GTC TG	CAC ACT GTG GAC AAA GTC TCT T
BIRC8	GCG CTC AGA AAG ACA CTA CAG	CCT CTT GCA GAC GCC TTA GC
BOK	GTC TTC GCT GCG GAG ATC AT	CAT TCC GAT ATA CGC TGG GAC
CARD18 (ICEBERG)	TGG GTG CAG GCA CAA TAA ATG	TTG AGG CAA GTT GAG GGT CTT
CARD6	GAG CAC GTT GGA TAT GAT GGT G	GGT CCA CTT ACA TCC TCG ATC TA
CARD9	CCC TCA CGC ATC ACA CCT TAC	GCA CAC CCA CTT TCC GTT TG
CASP10	TAG GAT TGG TCC CCA ACA AGA	GAG AAA CCC TTT GTC GGG TGG
CASP2	AGC TGT TGT TGA GCG AAT TGT	AGC AAG TTG AGG AGT TCC ACA
CASP4	CAA GAG AAG CAA CGT ATG GCA	AGG CAG ATG GTC AAA CTC TGT A
CASP6	CAC CAA CAT AAC TGA GGT GGA TG	AGG AGG AGC CAT ATT TTC CCA
CASP7	CGG TCC TCG TTT GTA CCG TC	CGC CCA TAC CTG TCA CTT TAT CA
CASP8AP2	ACA CAG TCG AGT AGA CTC TCA AA	AGG AAG TGA TGC TCG TTC AGA
CRADD	CAT CAG ACC GGC AGA TTA ACC	GTT GGC CTT ACA GCG GTA GAT
DEDD	GGA GAC ATC AAT TCG CTA TGT GA	GCA ACA CAC CAC AGG ATA GTG
DEDD2	TGA AGG CAA AGT GAC CTG TGA	AGG CGT CCA GAT AGG AGA GC
FADD	GCT GGC TCG TCA GCT CAA A	ACT GTT GCG TTC TCC TTC TCT
FASLG	CTC CGA GAG TCT ACC AGC CA	TGG ACT TGC CTG TTA AAT GGG
GAPDH	AGA AGG CTG GGG CTC ATT TG	AGG GGC CAT CCA CAG TCT TC
HIP1	ACA CGC CAG AAC GTG CAT A	CAC TGC GTT GCT AGA CAG AG
MCL1	TGC TTC GGA AAC TGG ACA TCA	TAG CCA CAA AGG CAC CAA AAG
NAIP	AAG GGA TTT GTT GAC ATA ACG GG	CAG CCG TAG TTC TTC GTA AGC
NFKB2	ATG GAG AGT TGC TAC AAC CCA	CTG TTC CAC GAT CAC CAG GTA
PEA15	GGA GAG CCA CAA CAA GCT G	CCA TAG TGA GTA GGT CAG GAC G
PIDD	TCA GAG GAT TCG GAC GCA G	GTG AGT GCT CAG ACG CAA GAA
PYCARD	TGG ATG CTC TGT ACG GGA AG	CCA GGC TGG TGT GAA ACT GAA
REL	ACA TGG TAA TTT GAC GAC TGC T	GCT TCC CAA TCG TTC AAC ACA
RELA	ATG TGG AGA TCA TTG AGC AGC	CCT GGT CCT GTG TAG CCA TT
RELB	CAG CCT CGT GGG GAA AGA C	GCC CAG GTT GTT AAA ACT GTG C
**TNFRSF25**	CCG TCC AGT TGG TGG GTA AC	CCA TCA CGT CGT AGA GCT GC
TP53 (p53)	CAG CAC ATG ACG GAG GTT GT	TCA TCC AAA TAC TCC ACA CGC
**TRADD**	GCT GTT TGA GTT GCA TCC TAGC	CCG CAC TTC AGA TTT CGC A
WAF1(p21)	TGT CCG TCA GAA CCC ATG C	AAA GTC GAA GTT CCA TCG CTC
XIAP	ACC GTG CGG TGC TTT AGT T	TGC GTG GCA CTA TTT TCA AGA TA
